# Does early linear growth failure influence later school performance? A cohort study in Karonga district, northern Malawi

**DOI:** 10.1371/journal.pone.0200380

**Published:** 2018-11-05

**Authors:** Bindu S. Sunny, Bianca DeStavola, Albert Dube, Scotch Kondowe, Amelia C. Crampin, Judith R. Glynn

**Affiliations:** 1 Department of Infectious Diseases Epidemiology, Faculty of Epidemiology and Population Health, London School of Hygiene and Tropical Medicine, London, United Kingdom; 2 Population, Practice and Policy Programme UCL Great Ormond Street Institute of Child Health, London, United Kingdom; 3 Malawi Epidemiology and Intervention Research Unit, Chilumba, Malawi; 4 District Education Office, Karonga, Malawi; CUNY, UNITED STATES

## Abstract

**Introduction:**

Stunting or linear growth retardation in childhood is associated with delayed cognitive development due to related causes (malnutrition, illness, poor stimulation), which leads to poor school outcomes at later ages, although evidence of the association between the timing and persistence of stunting and school outcomes within the sub-Saharan African context is limited.

**Methods:**

Anthropometric data around birth (0–4 months), early (11–16 months) and late childhood (ages 4–8 years) along with school outcomes up until the age of 11 were analysed for a cohort of 1,044 respondents, born between 2002–2004 in Karonga district, northern Malawi. The schooling outcomes were age at school enrolment, grade repetition in Standard 1 and age-for-grade by age 11. Height-for-Age Z-scores (HAZ) and growth trajectories were examined as predictors, based on stunting (<-2SD HAZ) and on trajectories between early and late childhood (never stunted, improvers, decliners or persistently stunted). Multinomial and logistic regression were used to estimate the association between stunting/trajectories and schooling, adjusted for socioeconomic confounders.

**Results:**

The effects of stunting on schooling were evident in early childhood but were more pronounced in late childhood. Children who were stunted in early childhood (9.3%) were less likely to be underage at enrolment, more likely to repeat Standard 1 and were 2–3 times more likely to be overage for their grade by the age of 11, compared to their non-stunted peers. Those persistently stunted between early and late childhood (7.3%) faced the worst consequences on schooling, being three times as likely to enrol late and 3–5 times more likely to be overage for their grade by the age of 11, compared to those never stunted. Compared to improvers, those persistently stunted were three times as likely to be overage by two or more years by the age of 11, with no effect on enrolment or repetition.

**Conclusion:**

Our findings confirm the importance of early childhood stunting on schooling outcomes and suggest some mitigation by improvements in growth by the age of starting school. The nutritional and learning needs of those persistently stunted may need to be prioritised in future interventions.

## Introduction

Childhood linear growth failure or stunting is associated with delayed cognitive development and may have related causes that include chronic malnutrition, recurrent exposure to infection, illness and poor stimulation in the early years of life. In 2013, over a third of the global estimate of 161 million stunted children below the age of five were in Africa [1]. Stunting in early childhood is a marker for adverse influences on growth and development. The first 1000 days since conception, until 24 months when growth faltering usually plateaus [2], is critical for the development of physical, sensory, language and cognitive function, and reflects the period most sensitive to nutritional deficiencies, poor stimulation and social neglect, with severe effects on child development and adverse implications in later life [3]. Catch-up growth may happen but those who are stunted in the early years are more likely to be stunted through adulthood [4,5], with possible inter-generational effects of stunting on the growth and development of subsequent generations [6]. At the prenatal stage, poor maternal nutrition (low BMI) is an important risk factor for restricted foetal growth and low birth weight. Poverty, marked by inadequate access to water and sanitation systems, poor nutrition and susceptibility to gastro-intestinal infections and diarrhoea, is strongly associated with stunting in the early years [7]. Growth in early life is also the period for brain development and cognitive functioning [8], while growth (specifically weight gain >24 months) in later life is predictive of substantial weight gain and the increased risk of chronic diseases in adulthood [5].

Studies on malnutrition and child development in low and middle-income countries have shown that linear growth in the first two years of life is predictive of early (<24 months) and later physical [9] and cognitive development [10–12], loss in economic productivity [13] and increased risk of chronic diseases [7,14]. However recovery from growth delays in early years is possible and has been found to be associated with improvements in cognitive development [15–17] though the extent of this growth recovery, and its impact on overall development is not well understood.

Early stunting has been found to be linked with late enrolment in school, grade repetition and poor school achievement [5,13,18–22] though few studies have examined this relationship within the sub-Saharan African context within the past decade. A longitudinal five-country birth cohort study, including South Africa, on the effects of early malnutrition and schooling [23] showed that stunting at the age of two was associated with delayed school enrolment, a greater chance of repeating at least one grade and fewer years spent in school. In rural South Africa and in Tanzania, children who were stunted were more likely to enrol late in school, repeat more grades [24] and complete fewer years of school [25]. Alderman et al’s [26] study in three resettlement areas in rural Zimbabwe showed that a 1-SD improvement in height-for-age at age 3 was associated with an earlier age at starting school, an additional grade of schooling, and improved height in adolescence. However, these studies do not provide a comparative effect of childhood growth and growth improvements on school performance and progression, within the sub-Saharan African context.

This study looks at the relationship between linear growth failure or stunting at birth (0-4months), early (11-16nths) and late childhood (4–8 years) on school outcomes, specifically age at enrolment in school, grade repetition in Standard 1, and progression (age-for-grade) by age 11. We also explore whether improvement in growth between early and late childhood influences school outcomes.

## Methods

Continuous birth registration was set up as part of the baseline census for a demographic surveillance carried out between 2002 and 2004 in the southern part of Karonga district, in northern Malawi. Trained staff collected anthropometric data during the first visit after birth, which was usually within 2–6 weeks. Repeat anthropometry measures were collected during a follow-up visit after one year. Anthropometric data were also collected in later survey rounds on all children under the age of 10 between 2008–2011, so data were available for the 2002–4 birth cohort at ages 4–8. For those measured more than once between 2008–11 the earliest record was used. Socio-economic and schooling histories were collected in the original census and updated annually from 2007 to 2015.

Routine training was provided to staff prior to collecting anthropometric data using methods recommended by the USAID’s Food and Nutrition Technical Assistance(FANTA) project[27]. For children below age 2, recumbent length was measured using a SECA210 polyurethane plastic measuring mat (with 0.5mm increments) while weight was measured using a spring scale (100g increments). Height of children older than two years was measured using the Leicester height measure. Maternal malnutrition, measured by the mother’s mid-upper arm circumference (MUAC), is a determinant of foetal growth restriction and early growth faltering [7,28]. In this study, MUAC was measured using a steel tape (1mm increments) and a cut-off of <21cm was used to define maternal malnutrition, as used previously in the same setting [29].

Early and later linear growth failure or stunting was defined as the Height-for-Age Z-score (HAZ) < -2 SD (termed as moderate/severe stunting) based on the WHO growth references for children below and above age 5[30,31]. The Z-score represents the difference in a child’s height from the median height of children within the reference population (at a given age and sex), divided by the standard deviation of the reference population. Growth trajectories between early and late childhood were defined as being never stunted, improvers (stunted in early childhood but not stunted in late childhood), decliners (not stunted in early childhood but stunted in late childhood), or persistently stunted (stunted in early and late childhood).

In Malawi, primary education is free and is for eight grades, with the official age of entry being 6 years. With the introduction of free primary education in Malawi in 1994, enrolment is nearly universal, though school quality is poor, with frequent grade repetitions and students progressing slowly through school[32]. Under or over age enrolment is possible. Household poverty, long distances to school and perceptions of school readiness may prompt parents to delay enrolment[33–35]. However, parents may also enrol children at an earlier age, to allow younger children to accompany their older siblings to school; to provide a head-start in school; or to optimise free child-care provision in school while parents work[33]. In our analyses, those who enrolled in school prior to or after the official age of entry of 6 were categorised as being underage or overage at enrolment. Age-for-grade is the number of years a child is ahead/behind in class based on the official age-for-grade (Age-for-Grade = Current Age-Current Grade-5) and provides a cumulative measure of school performance irrespective of the highest grade achieved. Given the follow-up time available for this cohort, the analyses focuses on age-for-grade at age 11, which is the age up until when most respondents were seen. The effects of stunting on grade repetition in Standard 1 is also examined.

Principal Component Analysis (PCA) was used to estimate relative household wealth at birth using data on dwelling characteristics (quality of walls, roof), ownership of consumer durables (clock, mosquito nets and bank account), and access to utilities (water, electricity). Categorical variables were made into dummy binary variables, while continuous variables (number of mosquito nets owned by a household) were re-scaled to have mean as zero and standard deviation 1 [36,37]. The first component explained 36% of the variation between households. The household wealth score was divided into thirds (most to least poor). Data on household assets collected between 2007–2011 were also used to construct asset indices for the follow-up period (early and late childhood) using PCA. Variables selected for inclusion in the asset index (bicycle, radio, oxcart, clock, mattress, bed and chair) were based on what was consistently available across all household survey rounds.

Data on parental educational qualifications were collected during the socio-economic surveys under the assumption that education levels remained unchanged since the child’s birth. A few other variables, including season at birth, mother’s age at birth, mother’s MUAC, birth order, were initially explored but omitted from the final analysis, as they did not appear to confound the relationships. Maternal height was not included because it can have a direct effect on foetal growth [9] and we wanted the growth measure to include any pre-natal growth deficit. Father’s height was explored as a possible confounder. Logistic regression was used to conduct the analysis for the grade repetition outcome. Multinomial logistic regression was used for the analyses on age at enrolment and age-for-grade at age 11. Significance of the relationship between stunting and grade repetition was assessed by performing a Wald test of each estimated OR being equal to 1. For the analyses of outcomes age at enrolment and age-for-grade at age 11 that were modelled using multinomial logistic regressions, Wald tests for the ORs for each level of outcome being equal to 1 were performed. In each case, we refer to these as tests for heterogeneity as these capture whether stunting at each age-interval has different odds of each outcome level.

Ethics approval for the study and the consent procedures were obtained from the National Health Sciences Research Committee in Malawi and the Research Ethics Committee of the London School of Hygiene and Tropical Medicine. Permissions to conduct the study were also granted by the traditional authorities, village headmen and traditional advisers in the study catchment area. For the demographic surveillance (which included schooling level recording and anthropometry at birth) the research purpose of surveillance was explained to each household. Verbal consent was sought (as was the norm for demographic surveillance studies at that time) and recorded by staff in the field register, along with any refusals to participate. Only those participants who consented to participate were included in the study. For the anthropometry study written informed consent was sought from the parent/guardian of each child.

## Results

**Fig 1:**
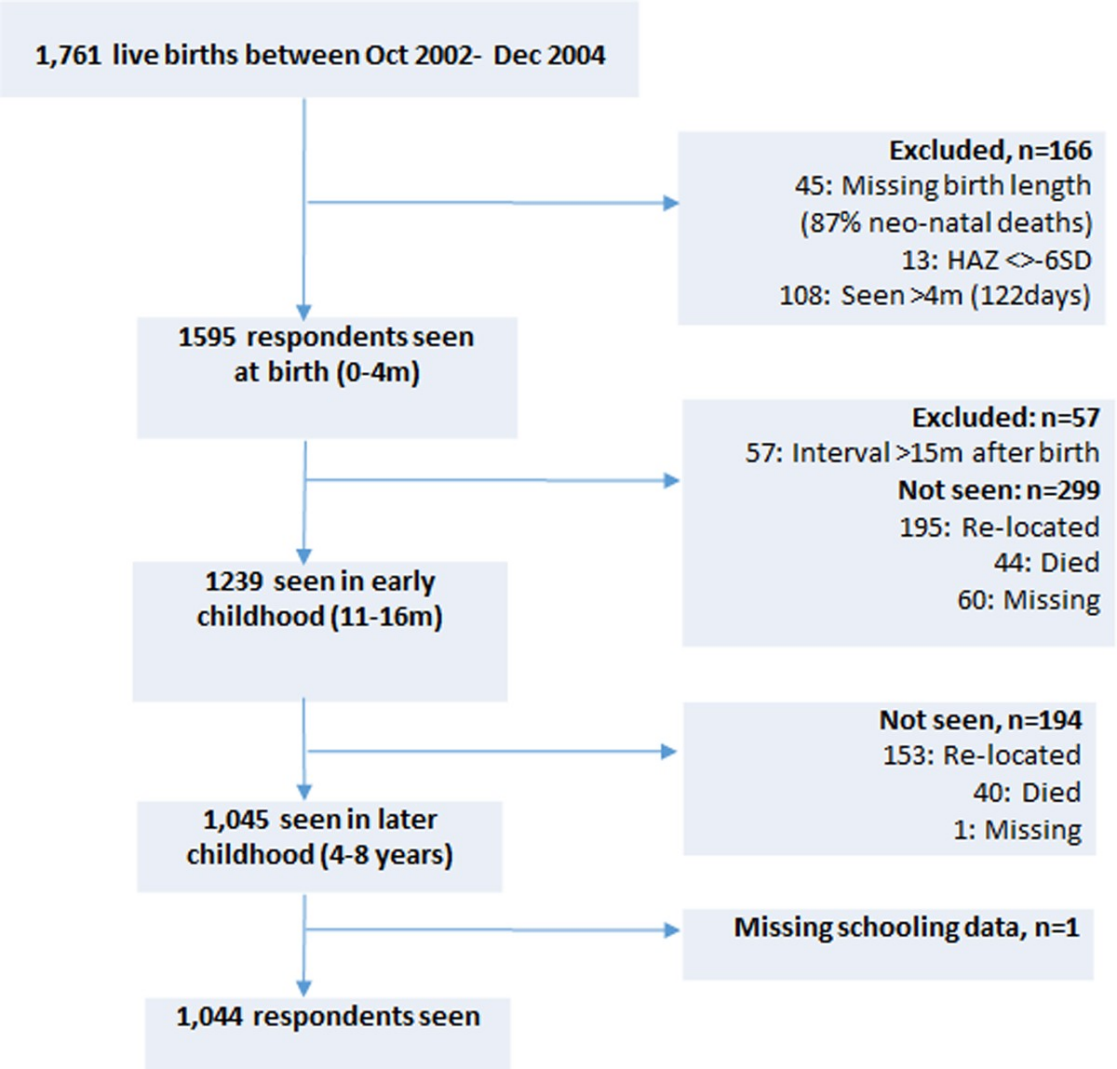
Study flowchart.

1,761 live births were recorded between October 2002 and December 2004 (Fig 1). Of these, 1595 (91%) respondents seen within the first four months of birth had data available on birth length. Those with missing data on birth lengths (n = 45) were mostly on account of neonatal deaths (87%) and outmigration from the surveillance area. 1239 (78%) of the remaining respondents were seen again in early childhood (11–16 months) within an interval not exceeding 15 months since birth. 1045 had anthropometry again between ages 4 and 8 years, of whom one had missing data in schooling (Fig 1). Complete case analysis was carried out: 5% had missing data on confounders for the school enrolment analysis, leaving 988 respondents. Data were available on grade repetition in standard 1 for 828 and on grade at age 11 for 789.

Table 1 examines the differences between groups lost to follow-up, those with incomplete data and those finally included in the analyses. Those with incomplete data were shortest at birth, were born to shorter mothers and were from poorer households in comparison to those in other groups, although there were very few with missing data on confounders (n = 56 or 5.3%). Children lost to follow-up on account of re-location and those not seen at time of interview were not very different from those included in the final analysis.

**Table 1:** Attrition levels and characteristics (mean, SD, median) of study participants lost to follow-up, those with incomplete data and those included in the analyses.

Characteristics	Missing_1_ at Year 1	Missing_1_ between Years 4–8	Incomplete_2_	Included/Complete data_3_
	(n = 255)	(n = 154)	(n = 56)	(n = 988)
Birth HAZ (mean, SD)	-0.38(1.17)	-0.50(1.22)	-0.68(1.23)	-0.52(1.15)
Birth WAZ (mean, SD)	-0.42(1.17)	-0.46(1.17)	-0.46(1.12)	-0.47 (1.05)
Mother's height (median, IQR)	155.2(151.4–158.9)	NA	154.9(151.1–158.8)	155.7 (152–159.5)
Mother's age at birth (mean, SD)	25.10(6.29)	24.56(5.42)	25.78(6.11)	25.83(6.45)
Mother's Mid-upper Arm Circumference(MUAC) at birth (median, IQR) cm	24.5 (23–26)	23.74(20.38–16.91)	24.91(2.49)	24.5(23.2–26)
% from poorest households (first tertile)	31.0	27.5	40.7	34.9

1. Those lost to follow-up on account of re-location or missing at survey

2. Those with missing data on confounders

3. Those included in the final analyses

**Fig 2:**
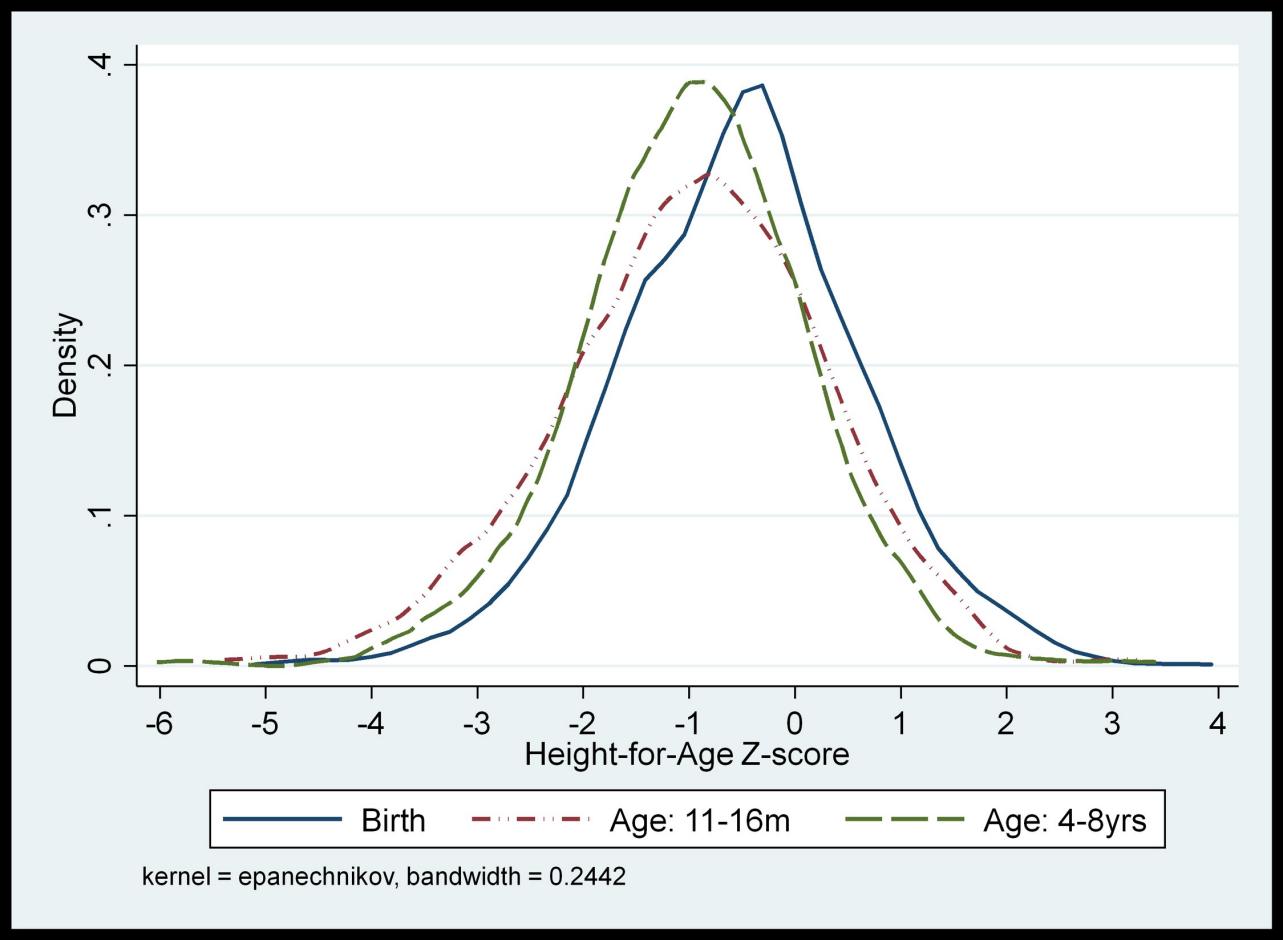
Distribution of HAZ at birth, early and late childhood.

Fig 2 shows the distributions of HAZ at birth, early and late childhood. The mean HAZ at birth lies closer to zero moving closer to -1SD through early and late childhood. There is an overall faltering of growth between birth and early childhood. Between early and later childhood the distribution of Z-scores narrows suggesting growth improvements among those shortest in early childhood with decline in growth among the tallest children. At baseline, children who were moderate-to-severely stunted (HAZ <-2) at birth had lower birth weight, were more likely to have been born in the hot/dry season, to mothers who were younger, shorter in stature and more likely to be malnourished shortly after birth (MUAC<21cm), than those not stunted at birth (Table 2). Stunting at birth was more prevalent among children from poorer families, with low (none, or less than full primary) parental education.

**Table 2:** Characteristics of respondents seen at birth (0-4m).

Characteristics	N	Stunted at birth	
		n	%
**Overall**	**1044**	**97**	**9.3**
**Sex**			
*Female*	500	38	7.6
*Male*	544	59	10.8
**Birth length (cm)**	1044		
*Mean*, *SD*		50.32 (2.89)	
*For non-stunted*, *Mean*, *SD*		53.44 (3.19)	
**Birth weight (kg)**	1044		
*Mean*, *SD*		3.80 (1.13)	
*For non-stunted*, *Mean*, *SD*		4.23 (3.23)	
**Mother's Education**			
*None/<Primary*	769	81	10.5
*At least Primary*	275	16	5.8
**Father's Education**			
*None/<Primary*	551	59	10.7
*At least Primary*	492	38	7.7
**Household asset index score**			
*Most poor-1*	356	39	10.9
*2*	340	38	9.2
*Least poor-3*	319	17	5.2
**Maternal malnutrition at birth**			
*No (MUAC> = 21cm)*	1005	87	8.6
*Yes (MUAC<21cm)*	39	10	25.6
**Season of birth**			
*Warm*, *rainy*	412	41	9.9
*Cool*, *dry*	433	32	7.3
*Hot*, *dry*	199	24	12.0
**Mother's Age at Birth**			
*Mean*, *SD*	1044	23.80(5.8)	
*For non-stunted*, *Mean*, *SD*		26.01(6.5)	
**Mother's Height**			
*Mean*, *SD*	1044	153.12(4.78)	
For non-stunted, Mean, SD		155.97(6.02)	

**Fig 3:**
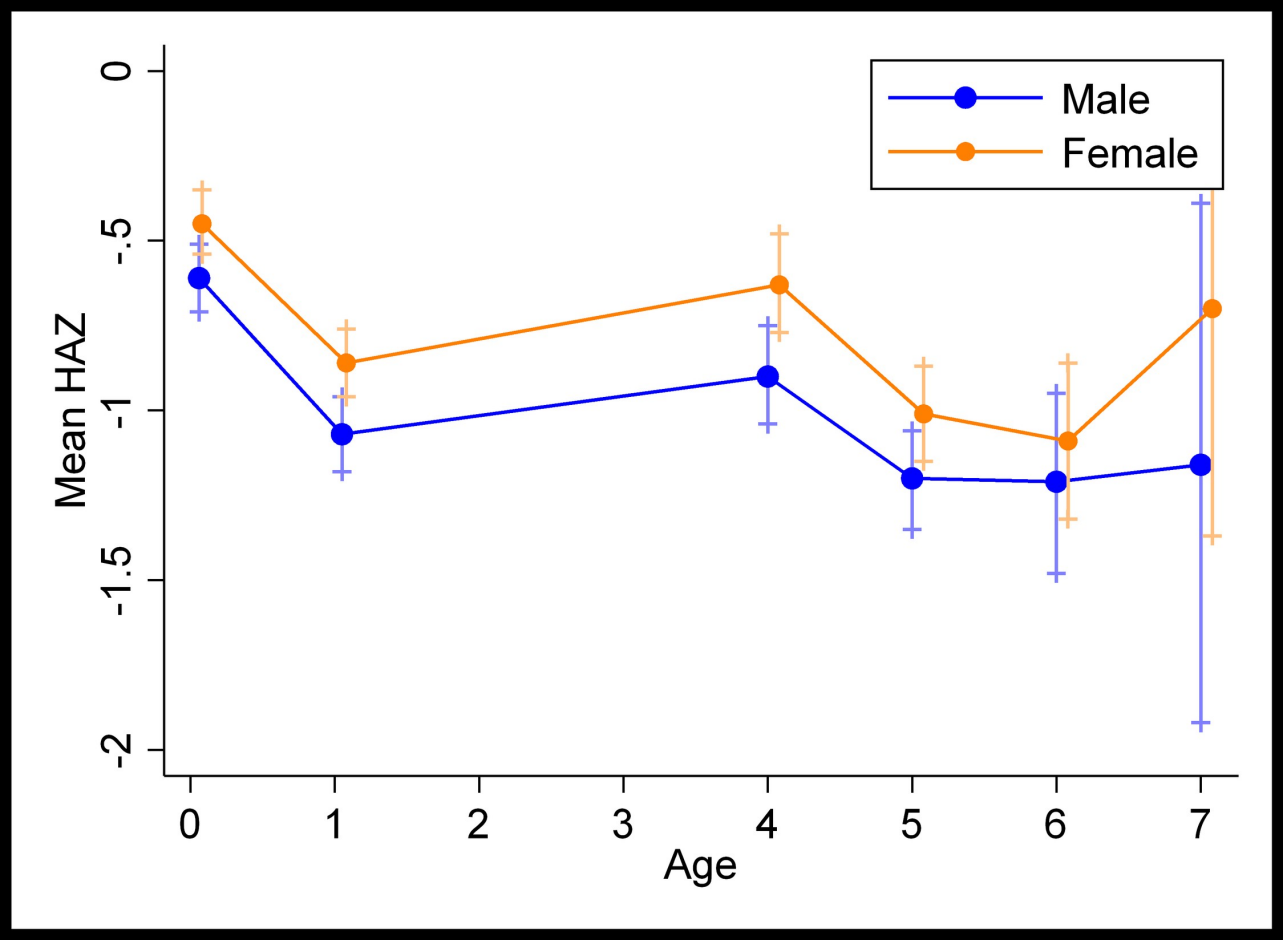
Distribution of the mean Height-for-Age Z-scores (and confidence intervals), by sex and age.

Fig 3 shows the distribution of the mean HAZ by age and sex. Growth faltered from birth until early childhood, improved until age 4 and then stabilised through late childhood, with fewer observations at age 7. On average, boys had lower Z-scores than girls. Overall stunting prevalence increased from 9% at birth to 20% in early childhood, with more boys (11% and 23%) than girls (7.7% and 15.6%) being stunted at both points. However, in late childhood, stunting prevalence fell to 15%, with boys continuing to show higher levels of stunting than girls (16% and 13%). As no evidence of interaction by sex was found on the associations between stunting and schooling outcomes, subsequent analyses are presented without disaggregating by sex.

Table 3 shows the association between stunting at different ages and schooling outcomes. Associations were weak with stunting at birth but were evident in early childhood, and were stronger and more pronounced in late childhood. Compared to those who were not stunted, those stunted in early childhood were 30% less likely (aOR = 0.66) to be underage at enrolment, and about twice as likely (aOR = 1.85) to be overage than on time at the point of entry, after controlling for potential confounders. Those stunted were twice as likely (aOR = 2.58) to also be at least two or more years overage-for-grade than underage/on time by the age of 11, compared to those who were not stunted. These effects were further magnified in late childhood with those stunted being around half as likely (aOR = 0.66) to be underage and twice (aOR = 2.82) as likely to be overage than on time at enrolment. Stunting in late childhood was also associated with being 2–4 times more likely to be overage than underage/on time for grade by the age of 11, even after adjusting for other socio-economic and other confounders (p<0.01). Effects of stunting on grade repetition in Standard 1 was weak at all three time-points. Associations with repetition and age-for-grade at 11 persisted after further adjustment for age at enrolment (S1 Table), showing that the associations were not explained by different enrolment ages.

**Table 3:** School outcomes associated with moderate/severe stunting at birth (0-4m), early (11-16m) and late childhood (4–8 years).

Outcomes	Birth (0-4m)					Early childhood (11-16m)					Late childhood (4-8yrs)				
	n/N	OR	CI	aOR_1_	CI	n/N	OR	CI	aOR_1_	CI	n/N	OR	CI	aOR_1_,_2_	CI
**Age at Enrolment (n = 991, 479 f, 512 m)**															
Under Age (<6)	36/492	0.67	0.43–1.05	0.7	0.45–1.11	75/492	0.64	0.46–0.89	0.66	0.47–0.92	41/491	0.44	0.29–0.65	0.47	0.31–0.71
On time(ref)	48/455	1		1		100/455	1		1		78/453	1		1	
Over Age (>6)	8/44	1.88	0.83–4.29	1.63	0.71–3.75	16/44	2.03	1.02–1.35	1.85	0.96–3.58	17/44	3.03	1.57–5.82	2.82	1.45–5.47
***Test for heterogeneity***		**p = 0.03**		**p = 0.10**			**p<0.01**		**p = 0.00**			**p<0.01**		**p<0.01**	
**Grade Repetition in Standard 1 (n = 830, 392 f, 438 m)**															
None(ref)	48/454	1		1		73/454	1		1		53/453	1		1	
1+ times	29/376	0.71	0.44–1.15	0.62	0.38–1.02	81/376	1.43	1.01–2.04	1.33	0.93–1.89	60/375	1.44	0.97–2.14	1.32	0.88–1.99
***Test for heterogeneity***		**p = 0.16**		**p = 0.06**			**p = 0.04**		**p = 0.12**			**p = 0.07**		**p = 0.17**	
**Age-for-Grade at Age 11 (n = 790, 368f, 422m)**															
Underage/On time(ref)	28/388	1		1		55/388	1		1		31/388	1		1	
1yr overage	24/239	1.44	0.81–2.54	1.25	0.69–2.25	55/239	1.81	1.20–2.74	1.68	1.10–2.57	39/239	2.25	1.36–3.71	2.21	1.32–3.72
2+yrs overage	24/163	2.22	1.24–3.96	1.77	0.95–3.28	52/163	2.84	1.83–4.39	2.58	1.63–4.10	45/162	4.43	2.68–7.32	4.18	2.44–7.16
***Test for heterogeneity***		**p = 0.03**		**p = 0.20**			**p<0.01**		**p<0.01**			**p<0.01**		**p<0.01**	

1. Adjusted for sex, father’s education, mother’s education, household asset index at birth

2. Adjusted for asset index around Age 4 (in late childhood only)

This table examines the association between stunting at three age-intervals (the main exposures, presented horizontally across three sets of columns) and age at enrolment, grade repetition and age-for-grade at Age 11 (which are the main outcomes, presented vertically, along three sets of rows). This organization allows comparison of estimated ORs across levels of stunting that are specific to three different periods. For example, children stunted in early childhood are almost twice as likely to be late at enrolment (“overage”) than to be on time, compared to those who were non-stunted. n/N shows those who had the outcome of interest as a proportion of those in the overall sample within that sub-group.

Compared to those who were never stunted, those stunted at some stage had worse school outcomes, with those persistently stunted facing the greatest disadvantage (Table 4). Being persistently stunted was strongly associated with later age at enrolment and with being overage for grade at age 11 even after adjusting for confounders. Associations with school outcomes among those who caught-up (“improvers”) and those who declined in growth status were similar in direction but showed weaker evidence of effect.

**Table 4:** Compared to those never stunted, effect on school outcomes for children with varying growth trajectories (improvers, decliners or with persistent stunting) from early to later childhood (4-8yrs).

Outcomes	Improvers					Decliners					Persistently stunted				
	n/N	OR	CI	aOR_1_	CI	n/N	OR	CI	aOR_1_	CI	n/N	OR	CI	aOR_1_	CI
**Age at Enrolment (n = 988)**															
Under Age (<6)	50/491	0.64	0.43–0.96	0.65	0.43–0.98	17/491	0.34	0.19–0.62	0.35	0.19–0.64	24/491	0.48	0.28–0.82	0.54	0.31–0.92
On time(ref)	61/453	1		1		39/453	1		1		39/453	1		1	
Over Age (>6)	7/44	1.8	0.73–4.45	1.64	0.66–4.09	8/44	3.22	1.33–7.80	3.07	1.26–7.51	9/44	3.62	1.54–8.51	3.22	1.35–7.68
*Test for heterogeneity*: *Crude OR*: *p<0*.*01*, *Adjusted OR*: *p = <0*.*01*															
**Grade Repetition in Standard 1 (n = 828)**															
None(ref)	48/453	1		1		28/453	1		1		25/453	1		1	
1+times	47/375	1.29	0.83–1.98	1.19	0.77–1.85	27/375	1.27	0.73–2.20	1.19	0.68–2.09	33/375	1.73	1.01–2.99	1.54	0.89–2.67
*Test for heterogeneity*: *Crude OR*: *p = 0*.*16*, *Adjusted OR*: *p = 0*.*41*															
**Age-for-Grade at Age 11 (n = 789)**															
Underage/On time(ref)	43/388	1		1		19/388	1		1		12/388	1		1	
1yr overage	36/239	1.6	0.99–2.59	1.42	0.86–2.35	20/239	2.02	1.05–3.88	1.69	0.85–3.37	19/239	3.03	1.44–6.40	2.53	1.17–5.50
2+yrs overage	22/162	1.69	0.96–2.97	1.42	0.77–2.64	16/162	2.78	1.38–5.62	1.76	0.79–3.93	29/162	7.99	3.92–16.26	5.12	2.35–11.16
*Test for heterogeneity*: *Crude OR*: *p<0*.*01*, *Adjusted OR*: *p = 0*.*00*															

1 Adjusted for father's education, mother's education, household asset index at birth, sex and asset index around age 4

Table 5 examines the effect of persistent stunting on school outcomes, compared to those who had shown improvements in growth between early and late childhood. Compared to ‘improvers’, the risk of being overage for grade by the age of 11 for those persistently stunted was four-fold (p<0.01), even after adjusting for confounders, including HAZ in early childhood. Effects on enrolment and grade repetition were smaller with very weak statistical evidence of association.

**Table 5:** Compared to improvers, effect on school outcomes for children persistently stunted between early (11-16m) and late childhood (4-8yrs).

Outcomes	Persistently stunted				
	n/N	OR	CI	aOR_1_	CI
**Age at Enrolment (n = 190, Improvers: 118, Persistently stunted: 72)**					
Under Age (<6)	24/74	0.76	0.40–1.41	0.73	0.37–1.45
On time(ref)	39/100	1		1	
Over Age (>6)	9/16	2.01	0.69–5.84	1.75	0.56–5.51
*Test for heterogeneity*:	**p = 0.20**			**p = 0.33**	
**Grade Repetition in Std 1 (n = 153, Improvers: 95, Persistently stunted: 58)**					
None(ref)	25/73	1		1	
1+times	33/80	1.35	0.70–2.60	1.17	0.58–2.37
*Test for heterogeneity*:	**p = 0.37**			**p = 0.66**	
**Age-for-Grade at Age 11 (n = 161, Improvers: 101, Persistently stunted:60)**					
Underage/On time(ref)	12/55	1		1	
1yr overage	19/55	1.89	0.81–4.41	2.17	0.87–5.43
2+yrs overage	29/51	4.72	2.03–11.01	4.04	1.61–10.18
*Test for heterogeneity*:	**p = 0.00**			**p = 0.01**	

1 Adjusted for father's education, mother's education, HAZ in early childhood, household asset index at birth, sex and asset index around age 4

## Discussion

Stunting at 11–16 months and 4–8 years was associated with delayed enrolments and poor progression through school. No significant effects on schooling were observed for those stunted at birth. Those persistently stunted through early and late childhood faced the most severe consequences of schooling. They were almost three times as likely to enrol late in school, and were 2–5 times more likely to be overage for their grade by age 11, compared to those never stunted. Even improvers and decliners were likely to face negative school outcomes, though less than those persistently stunted. Those persistently stunted were more likely to be overage for grade by age 11, than those who experienced improved growth. The stronger associations with stunting at later ages than at younger ages, and the better schooling outcomes in those whose HAZ improved is consistent with later growth having an important role in improving school performance.

Stunting in the first two years of life has for long been known to be a vital marker for growth with apparently little scope for recovery in later years [3]. However, recent studies have shown that ‘windows of opportunity’ for catch-up growth exist beyond the age of 2 years as well as in early adolescence [38] with possible effects on later school outcomes. For example, findings from the Young Lives study project in Ethiopia, Peru, India and Vietnam showed that stunting between ages 8–15 years was associated with lower grade completion and poorer performance in a language and mathematics test [15]. In Guatemala, height at 36 months was associated with grade attainment and literacy and numeracy scores among children at 18 years of age[39]. Our study findings are consistent with the evidence that shows that growth in early and later childhood are important determinants of schooling outcomes.

Two broad pathways may underpin the mechanism through which growth retardation in childhood leads to poor school outcomes: the “neural” hypothesis and the “development” hypothesis. The neural hypothesis emphasises the importance of the timely development of the brain, which if inhibited within the first two years may have deleterious, possibly irreversible effects on cognitive development [40,41]. The development hypothesis stipulates that early growth retardation is linked to delays in motor-neuron development and the physical development of the child. Children who have delayed physical mobility may experience lower stimulation from self-exploration, play and social interaction with parents and carers [11,42,43] which is predictive of verbal competency by the age of five [10,44] and poor psychological functioning in late adolescence [45]. Being stunted is also associated with behavioural and conduct difficulties, being hyperactive, less vocal and attentive than non-stunted children [6,11]. Children who are physically smaller in stature and appear to be less alert, articulate and ready for school may be treated differently (by parents, society, schools) than those not stunted [33,46,47]. This may explain the later school start of stunted children in our study. Beasley et al observed that children in rural Tanzania were perceived to be ready to enrol in school based on their height and when they were “able to reach their arm over the top of their head and touch the ear on the opposite side” [47]. Further research to examine parental and societal perceptions and decision-making on school readiness would help understand this better.

There are a few limitations in our study. Firstly, height measurements in early childhood were only available around 11–16 months, which is short of the 24-month window when growth faltering is known to reach a nadir, prior to catch-up growth taking place. This could under-estimate the extent of growth faltering in early childhood and the true extent of growth improvements that follow, with subsequent effects on school outcomes. Using Height-for-Age Z-scores (HAZ) may also over-estimate the extent of growth improvements seen, as HAZ uses age and sex-specific standard deviations of height as the denominator, which tend to increase with age. The use of absolute Height-for-Age Differences (HAD) may be a better measure for future studies [48], though the reliability in using either measure is widely debated. The use of self-reported data on school outcomes (age at enrolment, age-for-grade) may be subject to social desirability bias or measurement error. However, this is limited given the longitudinal nature of the data, where any missing or inconsistent responses were cross-checked and corrected using current/previous year’s schooling data. Being part of a larger demographic surveillance site with a continuous registration of vital events (birth, death and migration) allows for accurate reporting of age within the cohort, especially among the younger population.

Our study may also be limited by omitted variable bias and issues of endogeneity of prior health status and schooling. Parents may equalize or exacerbate differences in investments on their children’s health and schooling based on their initial perceptions of a child’s heath status or their cognitive endowments. Socio-economic and behavioural factors that influence these decisions, like household allocation of resources, parents’ attitudes and decisions on resource allocations (food, money for school, allocation of work vis-à-vis school), were not available. Episodes of illness, especially diarrhoea, within the household during infancy and early childhood, and measures of home environment and cleanliness may be an important determinant of children’s nutritional status but may also provide a measure of vulnerability to recurrent illness and school absenteeism that has an effect on school performance over time. These factors would need to be accounted for in future studies to understand the true extent of the effect of nutrition on schooling.

The higher prevalence of male stunting is consistent with a systematic review that used DHS surveys from 10 countries in sub-Saharan Africa to confirm that stunting prevalence was indeed higher among boys than girls in the region; however, the reasons for this remain elusive [49]. As our study sample was followed only to age 11, we were unable to establish the longer-term associations of stunting on adolescence and schooling, including school dropout, which is rare before age 13 in this population.

## Conclusion

Stunting in early and late childhood was associated with poor school outcomes (late enrolment and poor progression through school). While policies and programmes that prioritise improvements in nutritional status of children in the first 1000 days since conception remain crucial, improving nutrition beyond age 2 may also be beneficial. Reversing growth faltering should reduce stunting in later years, with benefits that extend to not just immediate health but also schooling, economic productivity and a better life for generations to follow.

## Supporting information

S1 Table School outcomes associated with moderate/severe stunting at birth (0-4m), early (11-16m) and late childhood (4–8 years), including age at enrolment as a mediator. (DOCX)
