# Supplementary material for: Does early linear growth failure influence later school performance? A cohort study in Karonga district, northern Malawi
Source: PLoS One. 2018 Nov 5;13(11):e0200380. doi: 10.1371/journal.pone.0200380 (PMC6218019; doi:10.1371/journal.pone.0200380)
Supplement: S1 Table — (DOCX) [file pone.0200380.s001.docx]

| **S1 Table: School outcomes associated with moderate/severe stunting at birth (0-4m), early (11-16m) and late childhood (4-8 years), including age at enrolment as a mediator** | | | | | | | | | | | | | | | |
| --- | --- | --- | --- | --- | --- | --- | --- | --- | --- | --- | --- | --- | --- | --- | --- |
| **Outcomes** | **Birth (0-4m)** | | | | | **Early childhood (11-16m)** | | | | | **Late childhood (4-8yrs)** | | | | |
|  |  |  |  |  |  |  |  |  |  |  |  |  |  |  |  |
|  | **n/N** | **aOR1** | **CI** | **aOR1,3** | **CI** | **n/N** | **aOR1** | **CI** | **aOR3** | **CI** | **n/N** | **aOR1,2** | **CI** | **aOR1,2,3** | **CI** |
| **Grade Repetition in Standard 1 (n=830, 392 f, 438 m)** | | | | | | | | | | | | | | | |
| None(ref) | 48/454 | 1 |  | 1 |  | 73/454 | 1 |  | 1 |  | 53/453 | 1 |  | 1 |  |
| 1+ times | 29/376 | 0.71 | 0.44-1.15 | 0.67 | 0.41-1.10 | 81/376 | 1.43 | 1.01-2.04 | 1.44 | 1.00-2.07 | 60/375 | 1.44 | 0.97-2.14 | 1.56 | 1.02-2.38 |
| *Test for heterogeneity* |  | **p=0.16** | | **p=0.11** | |  | **p=0.04** | | **p=0.05** | |  | **p=0.06** | | **p=0.04** | |
| **Age-for-Grade at Age 11 (n=790, 368f, 422m)** | | | | | | | | | | | | | | | |
| Underage/On time(ref) | 28/388 | 1 |  | 1 |  | 55/388 | 1 |  | 1 |  | 31/388 | 1 |  | 1 |  |
| 1yr overage | 24/239 | 1.25 | 0.69-2.25 | 1.21 | 0.66-2.20 | 55/239 | 1.68 | 1.10-2.57 | 1.6 | 1.04-2.47 | 39/239 | 2.21 | 1.32-3.72 | 1.92 | 1.13-3.25 |
| 2+yrs overage | 24/163 | 1.77 | 0.95-3.28 | 1.55 | 0.80-2.98 | 52/163 | 2.58 | 1.63-4.10 | 2.3 | 1.42-3.72 | 45/162 | 4.18 | 2.44-7.16 | 2.95 | 1.68-5.18 |
| *Test for heterogeneity* |  | **p=0.20** | | **p=0.42** | |  | **p<0.01** | | **p=0.001** | |  | **p<0.01** | | **p=0.001** | |
| *1.Adjusted for father's education, mother's education, household asset index at birth and sex* | | | | | | | | | | | | | | | |
| *2. Adjusted for asset index around Age 4 (in late childhood only)* | | | | | | | | | | | | | | | |
| *3. Age at Enrolment* | | | | | | | | | | | | | | | |
